# Effect of pH on Cleavage of Glycogen by Vaginal Enzymes

**DOI:** 10.1371/journal.pone.0132646

**Published:** 2015-07-14

**Authors:** Greg T. Spear, Mary McKenna, Alan L. Landay, Hadijat Makinde, Bruce Hamaker, Audrey L. French, Byung-Hoo Lee

**Affiliations:** 1 Department of Immunology/Microbiology, Rush University Medical Center, Chicago, Illinois, United States of America; 2 Whistler Center for Carbohydrate Research, Department of Food Science, Purdue University, West Lafayette, Indiana, United States of America; 3 CORE Center of Cook County Health and Hospitals System, Department of Medicine, Rush University Medical Center, Chicago, Illinois, United States of America; 4 Department of Food Science and Biotechnology, College of BioNano Technology, Gachon University, Seongnam, Korea; Univ. of Texas Health Science Center at San Antonio, UNITED STATES

## Abstract

Glycogen expressed by the lower genital tract epithelium is believed to support *Lactobacillus* growth in vivo, although most genital isolates of *Lactobacillus* are not able to use glycogen as an energy source in vitro. We recently reported that α-amylase is present in the genital fluid of women and that it breaks down glycogen into small carbohydrates that support growth of lactobacilli. Since the pH of the lower genital tract can be very low, we determined how low pH affects glycogen processing by α-amylase. α-amylase in saliva degraded glycogen similarly at pH 6 and 7, but activity was reduced by 52% at pH 4. The glycogen degrading activity in nine genital samples from seven women showed a similar profile with an average reduction of more than 50% at pH 4. However, two samples collected from one woman at different times had a strikingly different pH profile with increased glycogen degradation at pH 4, 5 and 6 compared to pH 7. This second pH profile did not correlate with levels of human α-acid glucosidase or human intestinal maltase glucoamylase. High-performance anion-exchange chromatography showed that mostly maltose was produced from glycogen by samples with the second pH profile in contrast to genital α-amylase that yielded maltose, maltotriose and maltotetraose. These studies show that at low pH, α-amylase activity is reduced to low but detectable levels, which we speculate helps maintain *Lactobacillus* growth at a limited but sustained rate. Additionally, some women have a genital enzyme distinct from α-amylase with higher activity at low pH. Further studies are needed to determine the identity and distribution of this second enzyme, and whether its presence influences the makeup of genital microbiota.

## Introduction

The lower genital tract microbiota of many women is dominated by bacteria of the genus *Lactobacillus*. In those women, production of lactic acid by *Lactobacillus* acidifies the vaginal environment resulting in an acidic pH [1]. A vaginal fluid pH of <4.5 is often used clinically as a characteristic to determine if a woman has a “healthy” microbiota while a pH of >4.5 is characteristic of bacterial vaginosis [2]. In a study that compared genital pH with microbiota identified by high throughput sequencing, Ravel et al. [3] found that in women whose lower genital tract microbiota was dominated by *Lactobacillus*, the pH was typically 4–4.5 while in most of the women whose microbiota was not dominated by *Lactobacillus*, the pH was >5.5. The low pH created by *Lactobacillus* is thought to be the main mechanism by which colonization by this bacterium reduces susceptibility of women to acquiring sexually transmitted infections [4–6].

It has been recognized for some time that glycogen, produced by the genital epithelium, is an important source of carbohydrate for growth of *Lactobacillus* in the lower genital tract [7]. However, ostensibly paradoxically, glycogen cannot be used directly by most isolates of genital *Lactobacillus* when cultured in vitro [8–10]. Recently, we reported that α-amylase is present in the lower genital tract fluid of women and that this enzyme processes glycogen into dimers, trimers and tetramers of glucose (maltose, maltotriose and maltotetraose) that can be utilized in vitro by *Lactobacillus* [11]. While there are two forms of α-amylase in the body, salivary and pancreatic, salivary α-amylase appears to be the major form found in the lower genital tract [12].

Since recent studies indicated that α-amylase breakdown of glycogen is important for colonization and growth of *Lactobacillus* in the lower genital tract, and since the lower genital tract pH can range from neutral to very acidic, it is of interest to understand how changes in pH can affect genital enzymatic processing of glycogen. Therefore, in this study, we determined the effect of varying pH on glycogen digestion by both α-amylase in saliva and the enzymes present in lower genital tract fluid.

## Methods and Materials

### Genital fluid and saliva

Cervical-vaginal lavage (CVL) samples were obtained from women who had provided written informed consent by irrigation of the cervix with 10 mL of nonbacteriostatic sterile saline, followed by aspiration from the posterior fornix. The study was approved by the Cook County Health and Hospitals System Institutional Review Board. All procedures followed Department of Health and Human Service guidelines. All women reported not douching and not engaging in sex within the 48 hours before sample collection. Trichomonas and yeast were not detected at the time of sample collection as determined by wet mount and potassium hydroxide.

All eight women were of good health. Seven were African American and one was Caucasian. Ages of the subjects ranged from 34 to 61; 5 were premenopausal and 3 were postmenopausal.

Saliva was collected from a normal healthy adult donor, diluted 1:1 with saline, sterile filtered, aliquoted and frozen. Written informed consent was obtained from this donor and the study was approved by the Rush University Medical Center Institutional Review Board. All procedures followed Department of Health and Human Service guidelines.

### Assay for glycogen degradation

Stock solutions of buffered glycogen at either pH 4, 5, 6 or 7 were made by mixing 5 ml of 250 mM HEPES (Fisher Scientific, city state) or 5 ml of 100 mM lactic acid, with 20 ml of 10 mg/ml glycogen (oyster glycogen, Sigma Chemical Co), and 20 ml of phenol-red-free RPMI 1640 medium as a source of divalent cations (Lonza, Walkersville, MD). The pH of each stock solution was then adjusted with HCl and sterile filtered.

Genital fluid, saliva (diluted 1:1000 in saline) or saline as a negative control (5 μl) were mixed with the glycogen stock solution (45 μl) in tubes and incubated at 37°C for 120 minutes. After incubation, 10 μl was transferred to wells of a microtiter plate and the pH in the wells adjusted to 7 by addition of 43 μl of 250 mM HEPES (pH 7) and 30 μl of phenol-red-free RPMI-1640 medium.

Glycogen was detected by color development using a modification of a previously-described assay [13] which consisted of addition to each well of 50 μl acetic acid (1.7 M), 50 μl potassium iodate (0.1 N) and 50 μl of potassium iodide (0.1 N). Plates were agitated and after 15 min read at 565 nm. The amount of glycogen in wells was calculated based on a standard curve made from dilutions of glycogen.

#### Enzyme quantification

Both acid-α-glucosidase and intestinal maltase glucoamylase were measured by ELISA. Kits were obtained from MyBiosource (San Diego, CA).

### Quantification of bacteria by Polymerase Chain Reaction

Bacterium-specific quantitative PCR (qPCR) assays were performed on isolated CVL genomic DNA [14]. Each 20 μl qPCR reaction contained Supermix (Bio-Rad Hercules, CA), primers, probes (IDT, Coralville, IA), and 1–10 ng template DNA. Primers and probes for the bacteria were described previously [14,15]. Known quantities of 16S rRNA plasmid targets were used as standards [14,15].

#### Oligosaccharide Analysis by High-Performance Anion- Exchange Chromatography

A high-performance anion-exchange chromatography (HPAEC) system equipped with an electrochemical detector (Dionex, Sunnyvale, CA) was used to determine hydrolysis products generated from oyster glycogen incubated with vaginal fluids. The filtered (0.45 μm) samples were separated using a CarboPac PA-1 pellicular anion-exchange column (Dionex) with gradient elution from 100% eluent A (150 mM NaOH) to 100% eluent B (600 mM NaOAc in 150 mM NaOH) [16]. Glucose (G1), maltose (G2), maltotriose (G3), maltotetraose (G4), and maltoheptaose (G5) were run as linear maltooligosaccharide standard molecules.

## Results

### Effect of pH on glycogen degradation by salivary α-amylase

**Fig 1:**
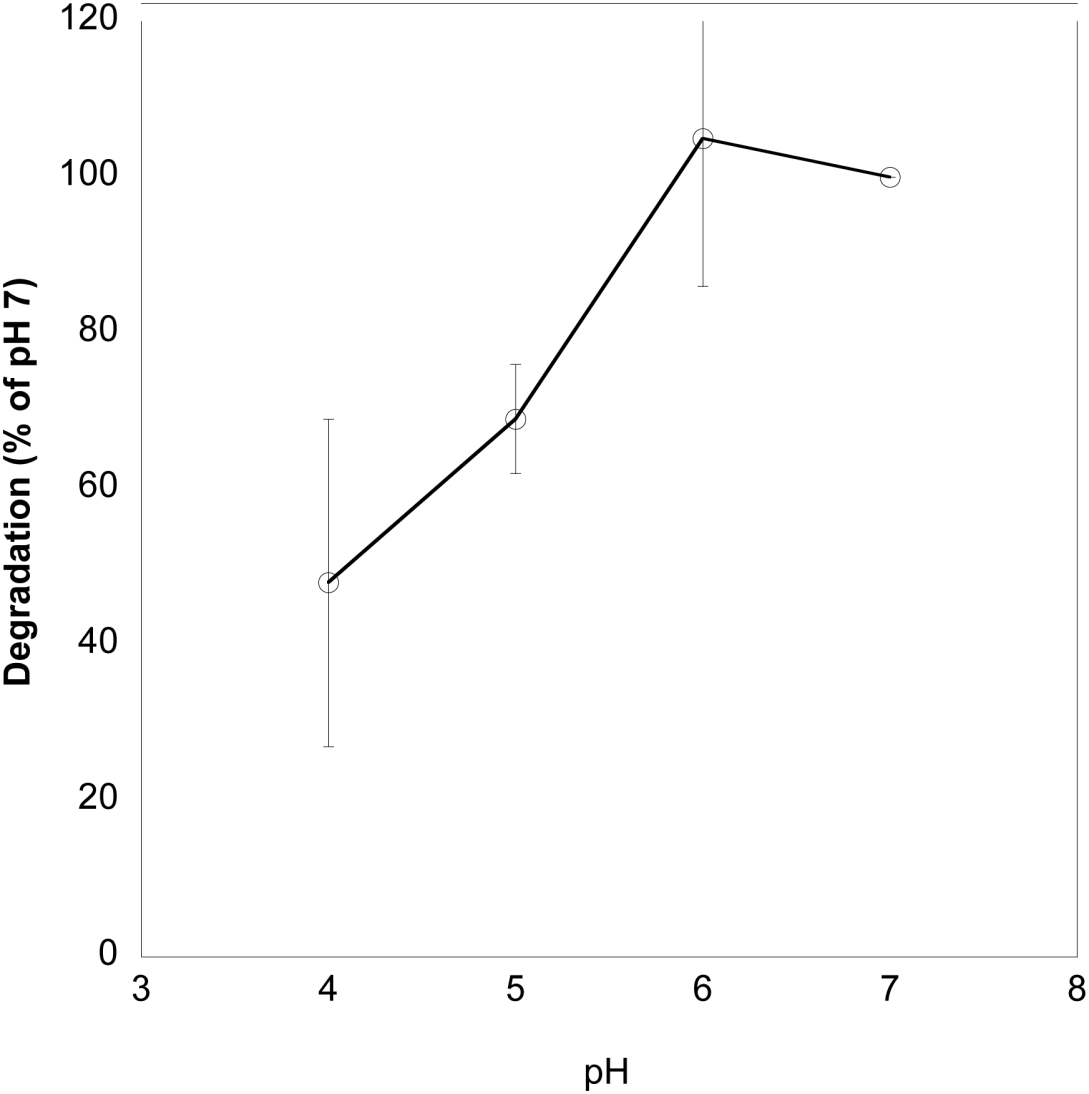
Effect of pH on glycogen degradation by salivary α-amylase. Saliva was collected from a normal donor and incubated with glycogen at pH 4–7. The percent of degradation of glycogen at pH 7 is shown on the y axis. Average of four experiments. Degradation at pH 4 was significantly different than that at pH 6 and 7 (p<0.05, Mann-Whitney test).

Since the pH of the genital tract can range from as low as 4 to neutral while the pH of saliva in the mouth is close to neutral [17], we first determined how α-amylase in saliva functions over a pH range. Saliva was collected from a normal adult donor and added to glycogen at either pH 4, 5, 6 or 7. At pH 6 and 7, salivary α-amylase caused the highest level of degradation (Fig 1). At pH 5 and 4, degradation was reduced to 69 and 48% respectively of the degradation found at pH 7.

### Effect of pH on glycogen degradation by lower genital tract fluids

**Fig 2:**
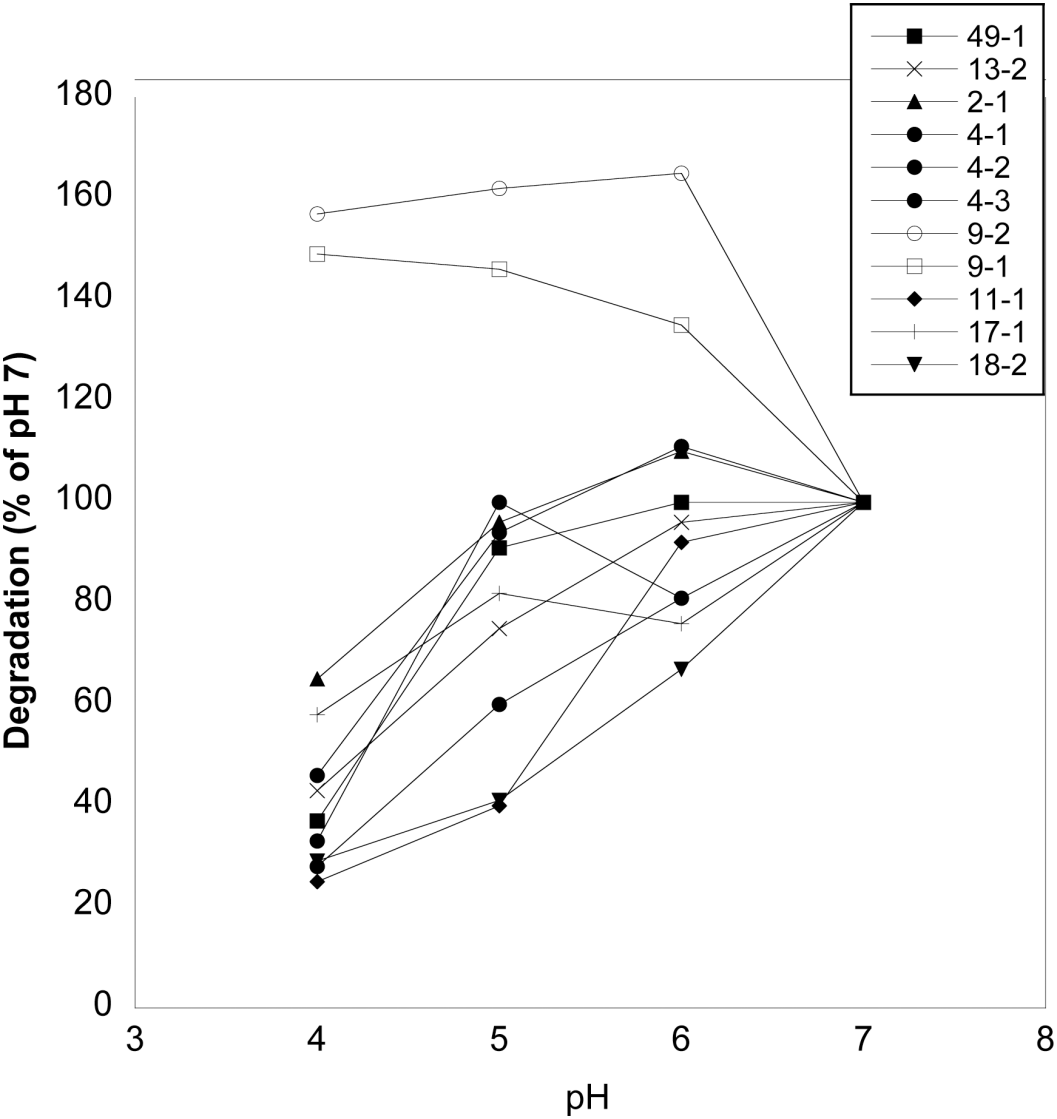
Effect of pH on glycogen degradation by lower genital tract fluids. Genital fluids were collected by lavage from eight donors. Two donors (subject 4 and subject 9) provided samples at multiple times (approximately one week apart). Lavage samples were incubated with glycogen at pH 4–7 and the percent of degradation of glycogen at pH 7 for each sample was calculated and is shown on the y axis. Each sample was run in two separate assays and the results averaged.

Lower genital tract fluid samples that were collected by lavage were also tested for degradation of glycogen at pH 4, 5, 6 and 7. Eleven samples from 8 different women were tested (two women provided samples at multiple time points). These samples were previously tested for α-amylase by ELISA and had levels that ranged from 1.7 to 10.9 units/ml [11]. Nine samples showed a pH sensitivity profile that was similar to α-amylase in saliva where maximal degradation of glycogen occurred at pH 7 and degradation was reduced, but not absent, at pH 4 (Fig 2). This included three samples from subject 4 (samples 4–1, 4–2 and 4–3) obtained at approximately one week intervals.

Interestingly, a sample taken from subject 9 (9–1) and a sample taken from the same woman one week later (9–2) showed a substantially different pattern of pH sensitivity (Fig 2) where degradation occurred at pH 7, but at lower pH, glycogen was degraded to a higher degree. Thus, these samples show two different patterns of pH sensitivity. In the type I pattern, salivary amylase and nine of the lower genital tract fluid samples degraded less glycogen at pH 4 than at pH 7. In type II, two samples from the subject 9 resulted in higher glycogen degradation at pH 4 than at pH 7.

Five of the samples with a type I pattern and sample 9–2 that had a type II pH sensitivity were also tested for glycogen degradation at pH 4 and pH 7 using a lactic acid-based buffer system to compare with the HEPES-buffered runs described above. All of the samples degraded glycogen at pH 7 in the lactic acid buffer. At pH 4, the 5 samples with type I pattern averaged 30% of the degradation observed at pH 7. This is in contrast to an average of 44% of pH 7 degradation when performed in HEPES buffer. Thus, low pH in a lactic acid-based buffer also reduced glycogen degradation. Subjecting sample 9–2 to pH 4 in lactic acid buffer increased degradation to 185% of that seen at pH 7 while in pH 4 HEPES buffer, the degradation was 157% of that seen at pH 7.

### Relationship of human enzymes, genital microbiota and genital pH to pH sensitivity of glycogen degradation

**Fig 3:**
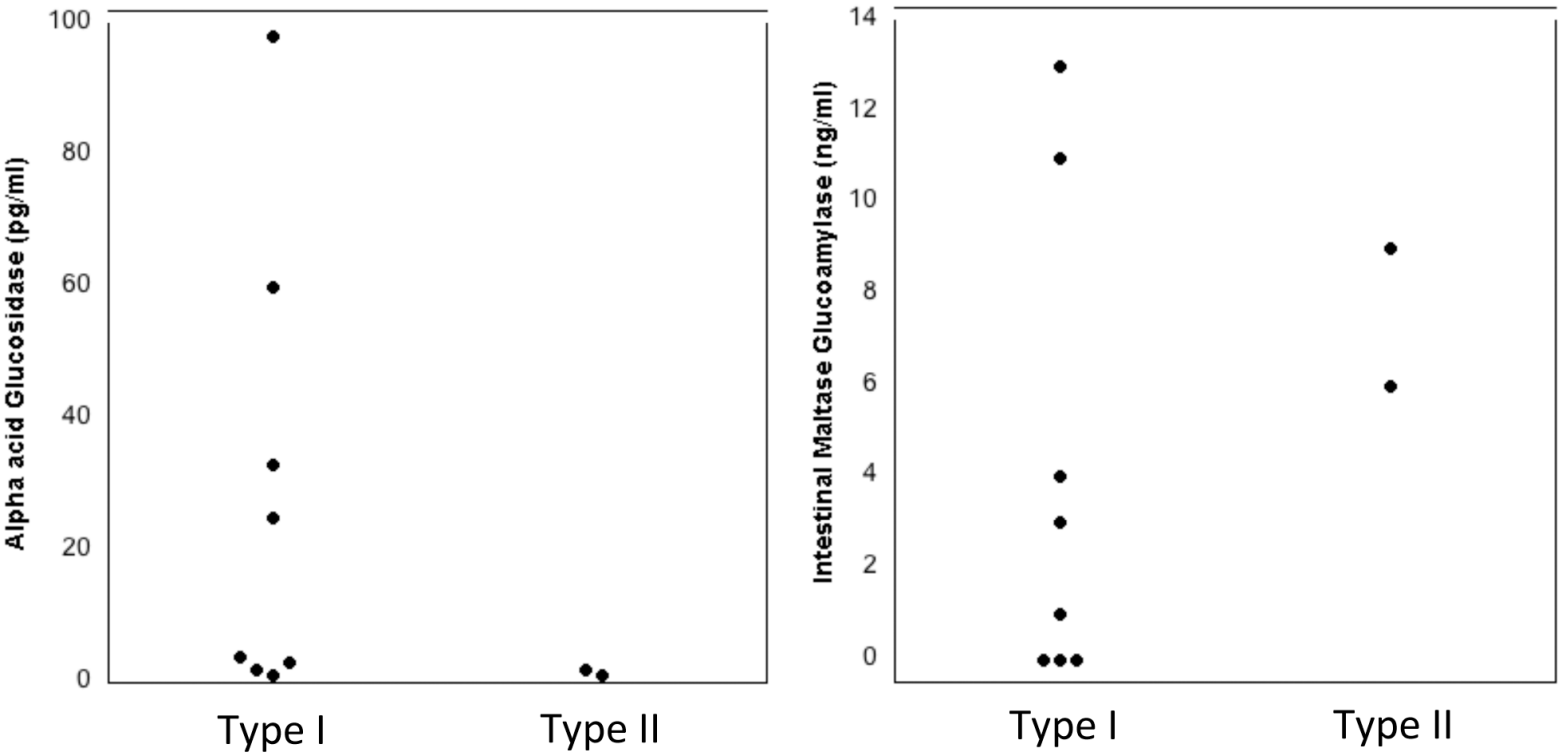
Carbohydrate-degrading enzymes in genital fluids. Levels of α-acid-glucosidase and intestinal maltase glucoamylase in genital fluid samples with Type I and Type II pH sensitivity were determined by ELISA. The average of triplicate ELISA wells is shown.

To determine if other human enzymes reported to be present in lower genital secretions could be responsible for type II pH sensitivity, we measured both acid-α-glucosidase [11] and intestinal maltase glucoamylase [12] in 8 of the 9 genital samples (a sufficient amount of sample 49–1 was not available). Acid-α-glucosidase was present in several of the samples from women with type I pH sensitivity, but was very low to absent in the two samples with type II pH sensitivity (Fig 3). Intestinal maltase glucoamylase was present in both samples with type II pH sensitivity but was also present at higher levels in two of the samples with type I pH sensitivity (Fig 3). Therefore, the presence of these two enzymes was not associated with type II pH sensitivity.

**Fig 4:**
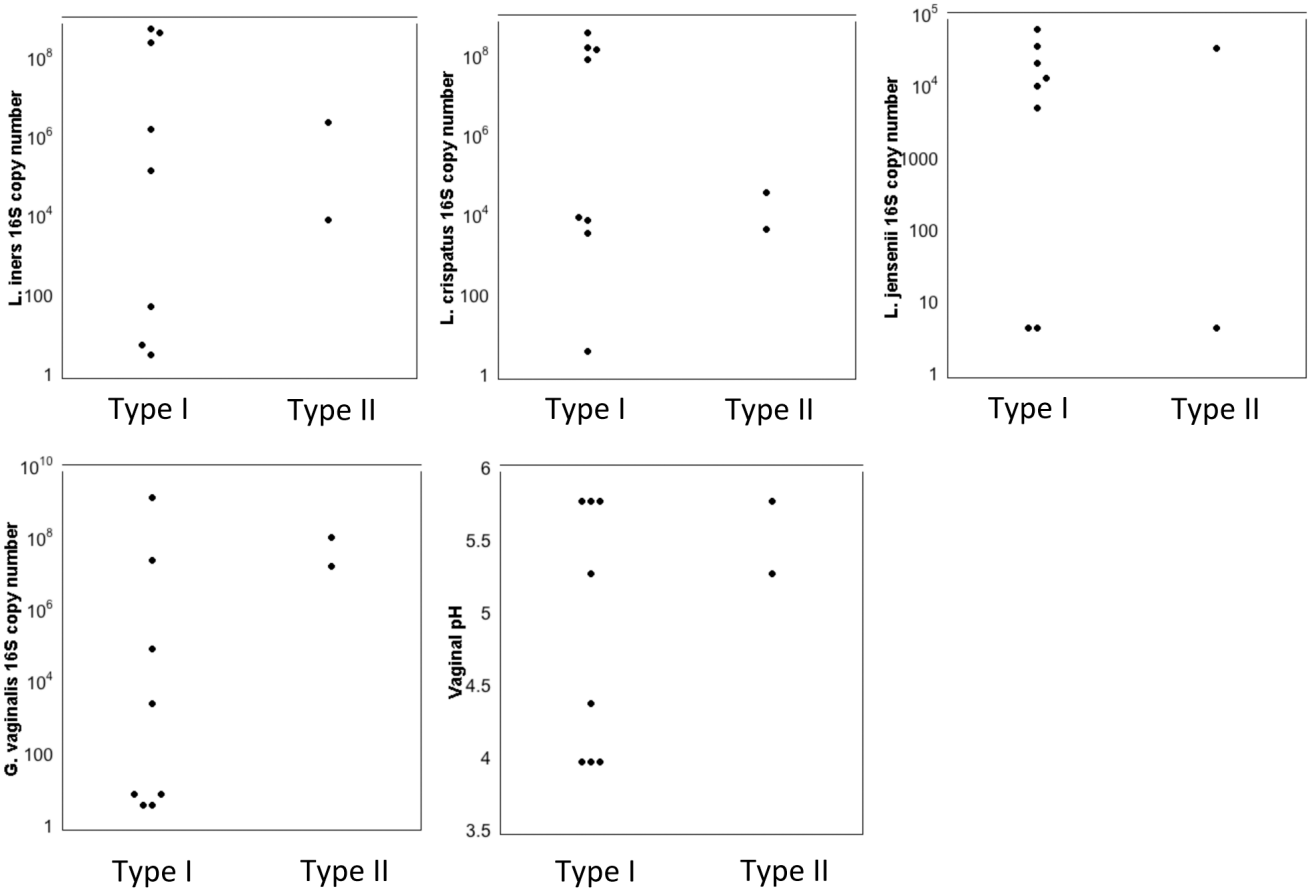
Types of commensal bacteria in genital samples. Levels of vaginal bacteria and vaginal pH corresponding to samples with Type I and Type II pH sensitivity. Bacteria were quantified in genital samples by species-specific real time PCR. Representative results from two assays are shown. Each assay was run in triplicate.

The microbiota in the lower genital tract and the vaginal pH of the women were also compared with type I and type II pH sensitivity of the enzymes. Neither genital levels of *L*. *iners*, *L*. *crispatus*, *L*. *jensenii*, *Gardnerella vaginalis* nor vaginal pH were consistently associated with either type I or type II pH sensitivity (Fig 4).

### Analysis of small sugars generated by genital enzymes

**Fig 5:**
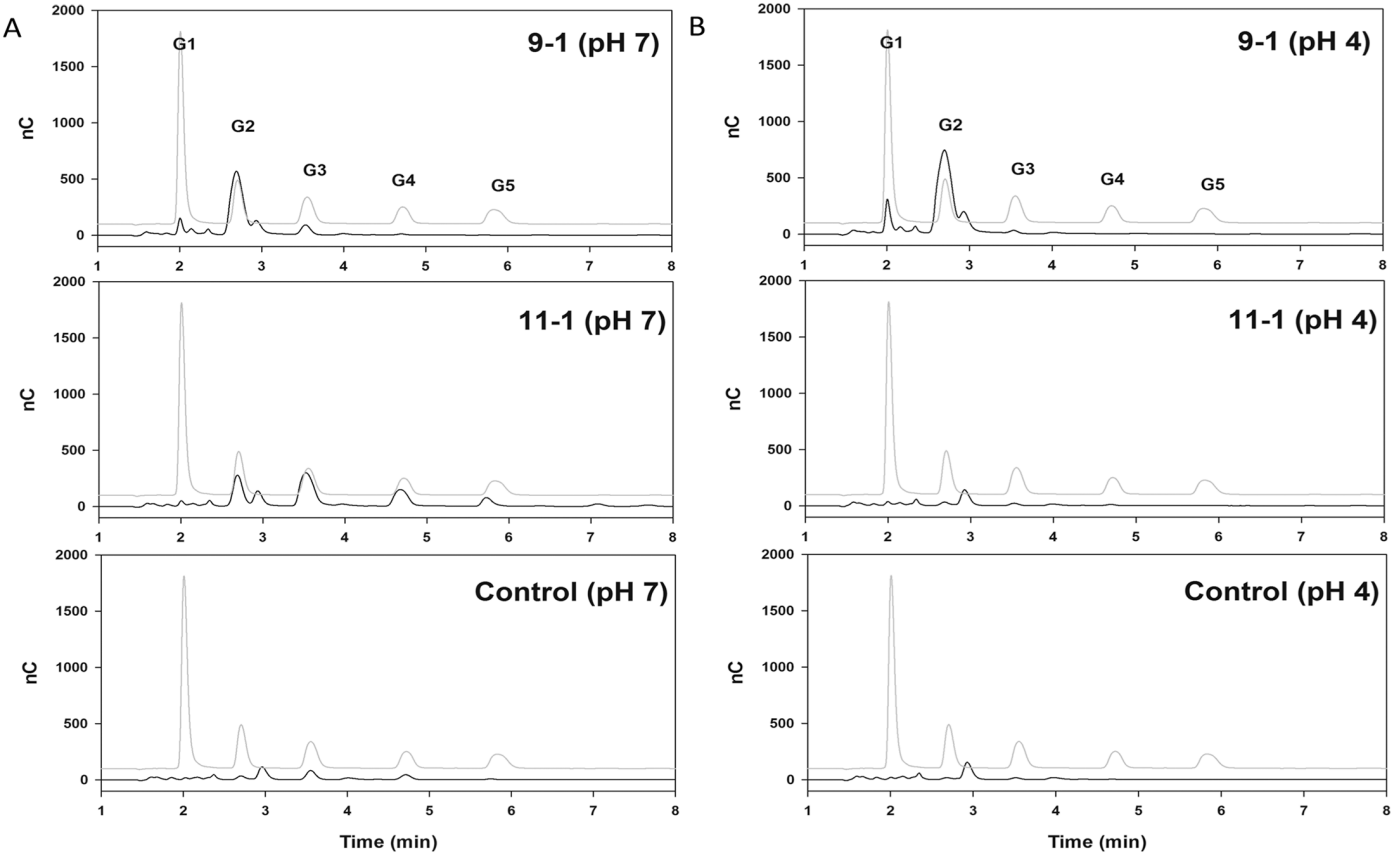
Size of small carbohydrates generated by glycogen degradation. HPAEC analysis of carbohydrates generated from glycogen by incubation with genital fluids (samples 9–1 or 11–1) or control (saline) at pH 7 (A) or pH 4 (B). The lighter trace on each graph represents the standards that were run including monomers (G1), dimers (G2), trimers (G3), tetramers (G4), and pentamers (G5). Runs were performed twice and representative results are shown.

To further characterize the type of enzymes associated with type I and type II pH sensitivity, high-performance anion-exchange chromatography was performed to identify the size of breakdown products generated from glycogen by the genital enzymes. Incubation of glycogen at pH 7 with a sample that exhibited the type I pH pattern (sample 11–1) resulted in small carbohydrates corresponding to maltose (G2), maltotriose (G3) and maltotetraose (G4) but with no glucose (G1) (Fig 5A). This pattern of small carbohydrates is consistent with α-amylase breakdown of glycogen as observed in our previous study [11]. When digestion with this sample was performed at pH 4, generation of small carbohydrates was substantially reduced (Fig 5B). Incubation of glycogen at pH 7 with a sample having the type II pH sensitivity (sample 9–1) resulted in mostly the G2 size and little if any G1, G3 or G4 (Fig 5a). At pH 4, this pattern was essentially unchanged except for an increase in the small amount of G1 (Fig 5b). The other sample with the type II pH sensitivity from the same woman, sample 9–2, resulted in essentially the same pattern of small carbohydrates as sample 9–1 at pH 4 and 7 (not shown).

## Discussion

Most lower genital tract isolates of *Lactobacillus* do not grow in media that contains glycogen as the only source of carbohydrates. However, our recent study shows that α-amylase is present in the lower genital tract, that this enzyme can process glycogen into small carbohydrates including maltose, maltotriose and maltotetraose, and that these small polymers of glucose are good sugar sources for growth of lactobacilli [11]. In the current study, we found that α-amylase activity is reduced, but not absent, at pH 4 and pH 5 when compared with pH 6–7. This could suggest that as *Lactobacillus* becomes the dominant vaginal bacterium and consequently lowers the vaginal pH, then processing of glycogen into small sugars by α-amylase is reduced. We speculate that this is a self-limiting step in the growth of genital lactobacilli which may help prevent rapid overgrowth and depletion of glycogen. In fact, in vitro cultures of some genital isolates of *Lactobacillus* can grow at exponential phase in medium with a pH as low as 3.9 if glucose is provided as a source of carbohydrate [18]. This suggests that a pH as low as 4.0 itself is not limiting for growth of genital lactobacilli, but our results suggest that the amount of small carbohydrates (e.g. maltose) could be limiting at pH 4.0 due to decreased processing of glycogen by α-amylase.

We recently reported that genital levels of cell-free glycogen are higher in women that have a low vaginal pH [19,20]. This could suggest that increased genital levels of glycogen support growth of *Lactobacillus*. However, the reduced α-amylase activity at low pH could contribute to increased levels of genital fluid glycogen since less breakdown of glycogen would occur. This introduces a chicken-and-egg type of conundrum; i.e., does high glycogen in the genital tract lead to the low vaginal pH through increased growth of lactobacilli or does the low vaginal pH lead to high glycogen due to decreased glycogen breakdown?

Vaginal sexual intercourse has been linked to decreased colonization by *Lactobacillus* [21]. Also, condom use is associated with increased colonization of *Lactobacillus* [22]. Further, it is established that semen neutralizes the low pH of the genital tract [23]. Therefore, our current study provides a potential explanation for these observations: that the effects of sexual intercourse and condom use on genital microbiota could be related to semen neutralizing the pH of vaginal fluid which would lead to increased degradation of glycogen by α-amylase, which in turn could lead to low glycogen levels and consequently reduced *Lactobacillus* growth.

In conclusion, these studies show that at low pH, α-amylase activity is reduced to lower but detectable levels which we speculate can continue to function more slowly in breakdown of glycogen into small polymers of glucose in the genital tract of women that have a genital tract microbiota dominated by *Lactobacillus*. However, these studies also show that some women have a genital enzyme that breaks down glycogen that appears distinct from α-amylase since it has higher activity at low pH. Further studies are needed to determine the identity and distribution of this second enzyme, and whether its presence influences the makeup of genital microbiota.
